# Profile and potential bioactivity of the miRNome and metabolome expressed in *Malva sylvestris* L. leaf and flower

**DOI:** 10.1186/s12870-023-04434-1

**Published:** 2023-09-19

**Authors:** Valentina Villani, Gabriele Di Marco, Federico Iacovelli, Daniele Pietrucci, Antonella Canini, Angelo Gismondi

**Affiliations:** 1 Department of Biology, Laboratory of Botany, University of Rome Tor Vergata, Via Della Ricerca Scientifica 1, Rome, 00133 Italy; 2 Department of Biology, PhD Program in Molecular and Cellular Biology, University of Rome Tor Vergata, Rome, Italy; 3 Department of Biology, Laboratory of Bioinformatics, University of Rome Tor Vergata, Via Della Ricerca Scientifica 1, Rome, 00133 Italy; 4 Department for Innovation in Biological, Agro-Food and Forest Systems (DIBAF), University of Tuscia, Via S.M. in Gradi N.4, 01100 Viterbo, Italy

**Keywords:** Common mallow, Leaf, Flower, MicroRNA, Anthocyanins, Next generation sequencing, Liquid chromatography, Cross-kingdom regulation

## Abstract

*Malva sylvestris* L. (common mallow) is a plant species widely used in phytotherapy and ethnobotanical practices since time immemorial. Characterizing the components of this herb might promote a better comprehension of its biological effects on the human body but also favour the identification of the molecular processes that occur in the plant tissues. Thus, in the present contribution, the scientific knowledge about the metabolomic profile of the common mallow was expanded. In particular, the phytocomplex of leaves and flowers from this botanical species and the extraction capacity of different concentrations of ethanol (i.e., 95%, 70%, 50%, and 0%; *v/v* in ddH_2_O) for it were investigated by spectrophotometric and chromatographic approaches. In detail, 95% ethanol extracts showed the worst capacity in isolating total phenols and flavonoids, while all the hydroalcoholic samples revealed a specific ability in purifying the anthocyanins. HPLC–DAD system detected and quantified 20 phenolic secondary metabolites, whose concentration in the several extracts depended on their own chemical nature and the percentage of ethanol used in the preparation. In addition, the stability of the purified phytochemicals after resuspension in pure ddH_2_O was also proved, considering a potential employment of them in biological/medical studies which include in vitro and in vivo experiments on mammalian models. Here, for the first time, the expressed miRNome in *M. sylvestris* was also defined by Next Generation Sequencing, revealing the presence of 33 microRNAs (miRNAs), 10 typical for leaves and 2 for flowers. Then, both plant and human putative mRNA targets for the detected miRNAs were predicted by bioinformatics analyses, with the aim to clarify the possible role of these small nucleic acids in the common mallow plant tissues and to try to understand if they could exert a potential cross-kingdom regulatory activity on the human health. Surprisingly, our investigations revealed that 19 miRNAs out of 33 were putatively able to modulate, in the plant cells, the expression of various chromosome scaffold proteins. In parallel, we found, in the human transcriptome, a total of 383 mRNAs involved in 5 fundamental mammalian cellular processes (i.e., apoptosis, senescence, cell-cycle, oxidative stress, and invasiveness) that theoretically could be bound and regulated by *M. sylvestris* miRNAs. The evidence collected in this work would suggest that the beneficial properties of the use of *M. sylvestris*, documented by the folk medicine, are probably linked to their content of miRNAs and not only to the action of phytochemicals (e.g., anthocyanins). This would open new perspectives about the possibility to develop gene therapies based on miRNAs isolated from medicinal plants, including *M. sylvestris*.

**Supplementary Information:**

The online version contains supplementary material available at 10.1186/s12870-023-04434-1.

## Introduction

Since ancient times, human civilizations have based their own health care on medicinal plants, making the latter one of the greatest heritages of modern mankind.

In the last decades, several pharmaceutical companies have focused their attention on plant secondary metabolites, especially polyphenols, due to their nutraceutical effect on mammalian systems. In detail, over 40% of the modern drugs consists of plant-derived substances or molecules synthesized considering the chemical structure of plant compounds [1].

Several studies have demonstrated the strong biological effects, including antioxidant and anti-inflammatory potential, of a wide range of phytochemicals that might represent natural raw substances by which developing new anticancer drugs [2].

*Malva sylvestris* L., also known as common mallow, is one of the most used species in traditional phytomedicine. It is a biennial, or perennial, erect herbaceous plant belonging to the Malvaceae family and native to Europe, Asia, and North Africa [3, 4]. Although the entire plant possesses therapeutic properties, leaves and flowers exhibit the most significant pharmacological activities, due to the presence of mucilage and flavonoids [5], useful for treating gastrointestinal disorders, respiratory diseases, and urological problems [6, 7]. In addition, some studies have demonstrated the capacity of mallow aqueous and hydroalcoholic extract to act as antinociceptive, reducing prostaglandin synthesis, oxidative stress levels, and the expression of proinflammatory factors [8, 9]. In general, *M. sylvestris* has been documented to be rich in anthocyanins. These secondary metabolites represent a widespread group of flavonoids, mainly responsible for flower and fruit staining in the range from black/dark blue to light pink. Indeed, several natural food colorants derive from these polyphenols [10]. In vitro experimental studies have proved that anthocyanin-rich extracts possess a potential capacity in promoting human health, thanks to their biological properties, including antioxidant, antiinflammatory, antidiabetic, antimicrobial, antineoplastic, and vasoprotective capacity [11–16], suggesting a key role at the expense of these flavonoids for the mallow. All this evidence has underlined the necessity to extend the scientific research towards clinical and toxicological aspects of *M. sylvestris*, in order to clarify the potential mechanism of action of its phytocomplex [3–17].

The phytocomplex is the set of chemical substances synthesized and accumulated in the tissues of a specific plant organism, living in a determinate environment. It includes both active and non-bioactive compounds, such as plant secondary metabolites (e.g., flavonoids, alkaloids, terpenes), sugars, and vitamins, which work in synergy. The phytocomplexes can exert their functions both on plant and animal systems, including humans. This potential would represent the scientific basis underlying the concept of ancient and modern phytomedicine/phytotherapy. Recently, one could hypothesise that even microRNAs (miRNAs, miRs) may be considered part of the phytocomplex. In this regard, in the last decade, various scholars have demonstrated and sustained the existence of a cross-kingdom regulation (CKR) mediated by plant miRNAs acquired through the diet [18–21]. Indeed, miRNAs have been classified as a group of small non-coding RNAs able to regulate the gene expression machinery, promoting degradation or translational arrest of their mRNA targets [22]. In support of CKR, it has been proved that the plant miRNAs can survive to heat cooking treatment (e.g., boiling), digestive processes (e.g., low pH), and enzyme activity, even persisting a few hours into the gastrointestinal tract before moving into bloodstream [23, 24]. The stability of these molecules would seem to be linked to a methyl group in position 2’-OH of the sugar residue at the 3’-terminal nucleotides, absent in animal miRNAs, which would protect them from 3’-uridylation and exonuclease degradation [22]. Moreover, the peculiar guanine-cytosine content, the association with Argonaute proteins, plant secondary metabolites, or polysaccharides, and the inclusion into exosomes are other features which could justify their extraordinary resistance [25]. To date, several papers have demonstrated the presence of miRNAs in medicinal and alimentary plants, opening the issue about the possibility that the beneficial impact of plant-derived extracts and foods on human health may be linked to their miRNA content and not only to the action of secondary metabolites [26, 27].

Considering the wide use of *M. sylvestris* in ethnobotanical practices and the absence in current literature of information about its miRNA profile, the main goal of the present research consisted in the molecular characterization of the phytocomplex obtained from the common mallow. In detail, to do it, the following aims were pursued: i) production of extracts from leaves and flowers of this plant species by maceration using different solvents; ii) biochemical typisation of the plant preparations through spectrophotometric approaches and High-Performance Liquid Chromatography associated to Diode Array Detector (HPLC–DAD); iii) analysis of the miRNome expressed in leaves and flowers of this medicinal plant by Next Generation Sequencing (NGS); iv) bioinformatic predictions of the putative plant and human mRNA targets for the detected miRNAs. Overall, our results amplified the scientific knowledge about the secondary metabolites present in this species and, for the first time, defined the list of miRNAs synthesized in *M. sylvestris* flowers and leaves, also trying to increase the comprehension of their potential biological functions both in the plant and on mammalian systems. In particular, the data we obtained about the latter aspect could open new perspectives on the possible miRNA-based CKR mechanism performed by this medicinal species on human health.

## Materials and methods

### Plant material and extraction procedures

*M. sylvestris* leaves and flowers were collected in June 2019 from twelve 4-months-old plants grown in the Botanical Gardens of Rome Tor Vergata and taxonomically identified by Prof. Antonella Canini. Portions of each sampled specimen deposited in the Herbarium of the Botanical Gardens of Rome Tor Vergata (botanical vouchers-codes: Ms1-12). The experimental research performed in this study complies with relevant institutional, national, and international guidelines and legislation; in particular, the collection of the plant material has been carried out in respect of the IUCN Policy Statement and has not involved any species at risk of extinction. The plant material was powdered, with mortar and pestle in the presence of liquid nitrogen and stored at -80 ˚C until the analysis. One hundred mg of leaf (L) and flower (F) powder were mixed with 1 mL of aqueous ethanolic solution (at 95% *v/v*, E; 70% *v/v*, 70WE; 50% *v/v*, 50WE; or 0% *v/v*, W) and left to macerate in agitation for 24 h at room temperature (RT), in the dark. After 20 min of centrifugation at 11,000 g, the samples were filtered with a 0.22 µm sterile membrane filter, obtaining pure extracts. For producing the respective aqueous extracts (henceforth aq), each original hydroalcoholic extract was totally desiccated at 30 ˚C by a vacuum dry evaporating system (Concentrator Plus, Eppendorf) and then resuspended in the same initial volume of pure ddH_2_O. Obviously, this last procedure was not performed on W samples.

### Total phenol content

Total phenol content was determined using Folin-Ciocalteu reagent (Sigma-Aldrich Co., St. Louis, MO, USA). Twenty-five µL of plant extract were added to 250 µL of Folin-Ciocalteu 0.2 N; after 5 min, the mixture was joined with 2 mL of 0.7 M Na_2_CO_3_ and incubated for 1 h in dark at room temperature (RT). The absorbance was measured at 760 nm and the results were expressed as micrograms of Gallic Acid equivalents per milligram of fresh material weight (μg GAE/mg FMW), using calibration curve prepared with increasing doses of gallic acid (0–25 mg/L).

### Total flavonoid content

The total amount of flavonoids was estimated by the aluminum trichloride solution (AlCl_3_) colorimetric method [28]. Twenty-five µL of plant extract were incubated, in the dark for 30 min at RT, with 5 μL of 10% AlCl_3_, 5 μL of CH_3_CO_2_K 1 M, 75 µL of methanol, and 140 µL of ddH_2_O. The absorbance was read at 415 nm. Results were expressed as micrograms of Quercetin equivalents per milligram of fresh material weight (μg QE/mg FMW), using a calibration curve adequately prepared with increasing concentrations of quercetin (0–25 mg/L).

### High Performance Liquid Chromatography-Diode Array Detector (HPLC–DAD) analysis

The chromatographic analysis of *M. sylvestris* samples was performed by an HPLC system equipped with SPD-M20A diode array detector (DAD, Shimadzu, Japan). The separation was achieved on a Kinetex C18 (2.6 µm × 2.1 mm × 10 mm) column at RT. For the detection of phenols and flavonoids, the mobile phase was composed of dH_2_O (solvent A) and isopropanol (solvent B). The gradient elution was set as follows: t_0min_ (A 85%, B 15%); t_20min_ (A 65%, B 35%); t_55min_ (A 10%, B 90%); t_68min_ (A 85%, B 15%); t_70min_ (end run). The flow rate was fixed at 0.95 mL/min, while the injection volume was of 10 µL. The detection was carried out by monitoring the eluate at 280 nm. The plant metabolites were identified and quantified by direct comparison with different concentrations of relative pure standards (Sigma-Aldrich Co., St. Louis, MO, USA), based on retention time, absorbance spectrum, and chromatographic peak area. The amount of each detected molecule was expressed as nanograms per milligram of fresh material weight (ng/mg FMW).

### Quantitation of anthocyanins

The content of specific anthocyanins was quantified using the pH differential spectrophotometric analysis according to the method of Giusti and Wrolstad [29]. In parallel, fifty µL of plant extract were mixed to 50 µL of 0.025 M potassium chloride buffer (pH 1.0) and 50 µL to 100 µL of 0.4 M sodium acetate buffer (pH 4.5). After incubation for 15 min, the absorbances of both solutions, for each sample, were measured at 505 nm (for quantifying Pelargonidin, PLG), 511 nm (for Peonidin, PEO), 510 nm (for Cyanidin, CYN), 519 nm (for Malvidine-3-glucoside, MAL3G), 523 nm (for Delphinidine, DEL), 520 nm (for Petunidin-3-glucoside, PTD3G) and 700 nm (as background), and used to solve the following formula:

$$\mathrm{A }= [({\mathrm{A}}_{x} - {\mathrm{A}}_{700}){\mathrm{pH}}_{1.0} - ({\mathrm{A}}_{x} - {\mathrm{A}}_{700}){\mathrm{pH}}_{4.5}]$$

where *x* is the specific wavelength at which each single anthocyanin absorbs. The anthocyanin content was calculated using the equation reported here:

$$Anth=\left(\mathrm A\;\times\mathrm{MW}\;\times\;\mathrm{dilution}\;\mathrm{factor}\;\times\;1000\right)\;/\;\left(\mathrm\varepsilon\;\times\;\mathrm l\right)$$

where A is the absorbance measured as previously reported, ε is the molar absorptivity of the measured anthocyanin (i.e., PLG ε = 15,600; PEO ε = 37,200; CYN ε = 24,600; MAL3G ε = 28,000; DEL ε = 34,700; PTD3G ε = 18,900), MW is the molecular weight of the measured anthocyanin (i.e., PLG MW = 271,24; PEO MW = 301,27; CYN MW = 287,24; MAL3G MW = 493,4; DEL MW = 303,24; PTD3G MW = 479,4), and l is the length of the cuvette. Results were expressed as micrograms of anthocyanin per milligram of fresh material weight (μg/mg FMW).

### MiRNA extraction, validation, and sequencing

MiRNAs were purified from *M. sylvestris* powdered flowers (i.e., F01, F02, F03) and leaves (i.e., LE01, LE02, LE03) using the mirPremier microRNA Isolation Kit (Sigma-Aldrich, St. Louis, USA), according to the manufacturers’ instructions. Concentration and purity of the miRNA extracts were estimated by spectrophotometric analysis (NanoDrop 2000, Termo-Fischer Scientific, USA). Then, 60 ng of miRNAs were retro transcribed to cDNA by the miScript II RT Kit (Qiagen, USA), following the guidelines of the producers. During this step, a synthetic spike-in control miRNA (UniSp6, EXIQON) was added to the reaction, to check the absence of nucleases during the procedures and the efficiency of cDNA synthesis and PCR amplifications. Thus, to verify the extraction method, qPCR assays were carried out using miRNA cDNA as template, as widely reported in Gismondi et al. [30], by an IQ5 thermocycler (Bio-Rad). In particular, the presence of UniSp6, ubiquitous plant miRNAs (miR159—miRBase accession number: MI0021329; miR397-5p – miRbase accession number: MIMAT0035795) and plant 5S rRNA (considered as positive controls; *Arabidopsis thaliana* GenBank: AB073495.1) was investigated, together with the incapacity of the amplification to occur in absence of template (Neg1) or primers (Neg2) in the reaction mixture. PCR products were fractionated by electrophoresis on an agarose gel (1%; w/v) containing 10 mg/mL ethidium bromide and using TAE (40 mM Tris; 1 mM EDTA; 20 mM acetic acid; pH 8.5) as running buffer. The amplicons were visualized and photographed under UV light (Gel Doc 2000 BIO-RAD). Once the isolation protocol was validated, as reported above, the pools of miRNAs were subjected to NGS analysis. Briefly, the library was prepared using QIAseq miRNA Library Kit (QIAGEN) and quantified by a Qubit 2.0 Fluorometer (Invitrogen). Then, NGS was carried out using the HiSeq 2500 platform (2 × 75 paired-end format; Illumina, San Diego, CA, USA). At least 80% of bases called with a quality score of 30 or higher. The datasets generated and analysed during the current study are available in the Sequence Read Archive (https://www.ncbi.nlm.nih.gov/sra; ID: PRJNA952515; submission: SUB13030104).

### MiRNA annotation from small RNA-seq data

To define *M. sylvestris* L. leaf and flower expressed miRNomes and quantify the level of the detected plant miRNAs, a genome-wide miRNA annotation method was applied using the small RNA-seq data obtained by NGS analysis. To perform this task, we exploited the *miR-PREFeR* pipeline [31], a fast and versatile tool used for profiling miRNA expression patterns, following the criteria recommended for plant microRNA annotation [32]. As *M. sylvestris* genome has not yet been assembled and registered in scientific databases, the genetic information available for *Gossypium raimondii* Ulbr.*,* a species belonging to the same family (Malvaceae) of common mallow, was downloaded in *fasta* format from NCBI (BioProject section; Taxonomy ID: 29,730; BioProject Accession: PRJNA171262 and ID: 171,262), and employed as a reference [33]. The quality of the raw reads from the small RNA-seq data obtained by NGS was evaluated with *FastQC* v.0.11.9 (https://www.bioinformatics.babraham.ac.uk/projects/fastqc/). All the *fastq* files were filtered to remove low-quality reads and adapters with *Trimmomatic* v.0.39 [34]. *M. sylvestris* miRNome has been identified taking advantage of the *miR-PREFeR* pipeline [31], a fast and versatile tool using expression patterns of miR and following the criteria for plant microRNA annotation to accurately predict plant miRs from samples of small RNA-seq data. The filtered *fastq* files were converted in a *fasta* format, characterized by a specific header containing the number of reads for each sequence as required by *miR-PREFeR* using the provided *process-reads-fasta.py* script, and aligned on the reference genome by a second script, *bowtie-align-reads.py*. The output of these steps consisted in a single *sam* alignment file, which was used as input for *miR-PREFeR* main code, while unmapped alignments were filtered using *SAMtools* [35]. The output consisted of a file containing the read counts for each mature and precursor miRNA sequence, both from leaf and flower samples. The mature sequences were aligned, using the *blastn* algorithm of BLAST, against the *Viridiplantae* section of miRBase [36], to search for their homologs in the Plant Kingdom.

### MiRNA data analysis

Small RNA-seq data were analyzed using the R statistical software (version 6.3), using the following functions: DESeq2, phyloseq, pheatmap, and ggplot2, with default parameters [37, 38]. To explore the variability of abundance in miRNAs between *M. sylvestris* leaf and flower samples, Principal Component Analysis (PCA) was carried out. Thus, the contribution of all miRNAs on each component was investigated per each sample. These data were statistically confirmed using the Principal Coordinate Analysis (PCoA, Bray–Curtis), the PERMANOVA test (number of permutations = 9999), and the DESeq2 test (False Discovery Rate *p*-value < 0.05). miRNAs identified in leaves and flowers without any statistically significance were considered ubiquitous for *M. sylvestris* tissues. The HEATMAP showing miRNA abundances was created through R software (ggPlot function).

### Prediction of miRNA putative plant and human targets and Gene Ontology (GO) enrichment analysis

PsRNAtarget, a plant small RNA target analysis server (http://plantgrn.noble.org/psRNA Targe t/) was used to identify all putative plant mRNA targets for the miRNAs detected in the common mallow. The research was performed by the function *submit small RNAs* in the *analysis section*: the sequence of each *M. sylvestris* miRNA was uploaded as query, while *G. raimondii* (Unigene—DFCI Gene Index-GORAGI, version 1 released on 2008) was selected as reference library, considering that the genome of this species was also employed as reference during the step of miRNA annotation. The prediction was carried out using the default scoring schema V2 (released on 2017). On the other hand, to predict the human transcripts putatively regulated by *M. sylvestris* miRNAs, an Support Vector Machine (SVM)-based classifier, trained using an experimentally validated set of miRNA-mRNA interactions developed in our previous publications [39, 40], was applied. In predicting the putative human targets, the analysis focused on the transcripts related to four categories of animal cell processes (i.e., apoptosis, cell cycle, redox state, and senescence). To better understand the main biological functions and networks potentially linked to the whole set of putative plant or human targets for all the detected miRNAs in leaves and flowers, the results obtained from the previous bioinformatics predictions were subjected to an enrichment analysis of Gene Ontology (GO) terms using the Metascape online tool v3.5.20230101 (http://metascape.org) [41], setting *A. thaliana* or *Homo sapiens* as input/analysis species according to the case.

### Statistics

All results were expressed as mean value ± standard deviation (s.d.) of different measurements obtained from independent experiments (n ≥ 3). Statistical significance was evaluated, through the Excel software, by the one-way ANOVA test and the post-hoc Lowest Standard Deviations test. *p*-values < 0.05 were considered statistically significant (*p*-values: * < 0.05; ** < 0.01; *** < 0.001).

## Results and discussion

The synthesis of secondary metabolites in plants depends on both endogenous (e.g., genetic) and exogenous (e.g., environmental) factors, as they are involved in the protection against biotic and abiotic stresses and in several steps of plant propagation [26]. Thus, the phytocomplex of each plant organism would represent a unique fingerprint able to distinguish it from another individual, although specimens belonging to the same taxon may show highly overlapping profiles. Similarly, the miRNome could be considered a distinctive molecular signature, whose composition can vary even among different tissues of the same plant. To justify this phenomenon, one should bear in mind that miRNAs carry out an important role in modulating gene expression, suggesting that each plant district induces the production of specific miRNAs at specific timings [42, 43].

Since ancient times, it has been understood that plant compounds exerted biological properties even on the human body, promoting the development of folk medicinal practices based on natural extracts. Surprisingly, as reported above, such type of CKR has also been recently linked to the activity of plant miRNAs. Therefore, investigating the metabolome and the miRNome of a plant can clarify the role of the respective components in the various tissues, favour the characterization of the species, and promote the comprehension of its potential beneficial effect on the human health. Taking into account all this evidence and the great ancient and modern phytotherapeutic role of the common mallow (whose molecular mechanisms have not been totally clarified yet), in this work the phytocomplex and the miRNA profile from *M. sylvestris* leaves and flowers were typified and analysed.

### Metabolome analysis

**Fig. 1:**
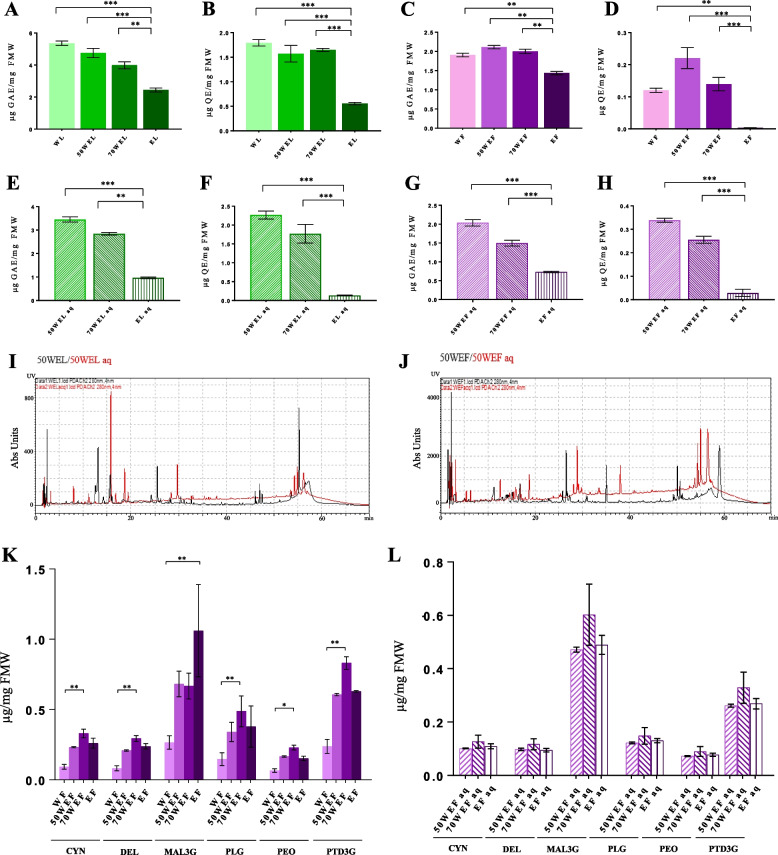
Biochemical analyses. **A-H** Spectrophotometric quantitation of phenols (panels **A**, **E**, **C**, and **G**) and flavonoids (panels **B**, **D**, **F**, and **H**) in the different pure (WL, 50WE, 70WE, and E) or aqueous (aq) extracts obtained from common mallow leaves (panels **A**, **B**, **E**, and **F**) and flowers (panels **C**, **D**, **G**, and **H**). Results are expressed as mean ± s.d. of four independent measurements (**p < 0.01; ***p < 0.001) and reported as µg of standard equivalent per mg of fresh material weight (FMW). **I-J)** HPLC–DAD profiles acquired at 280 nm. Representative chromatograms relative to 50WEL and 50WEF samples (in black) and their corresponding aqueous extracts (aq; in red) are shown in overlapping modality. **K-L)** Spectrophotometric characterization of the anthocyanins (Cyanidin, CYN; Delphinidine, DEL; Malvidine-3-glucoside, MAL3G; Pelargonidin, PLG; Peonidin, PEO; Petunidin-3-glucoside, PTD3G) from pure (panel **K**) and aqueous (aq; panel **L**) *M. sylvestris* flower extracts (WF, 50WEF, 70 WEF, EF). Results are expressed as mean ± s.d. of three independent measurements and reported as µg of standard equivalent per mg of fresh material weight (FMW) (*p < 0.05; **p < 0.01)

In the present experiment, different types of solvents (i.e., 95%, 70%, and 50% hydroalcoholic or aqueous solutions) were employed to isolate mallow phytocomplex. Indeed, one of the aims of this research was also to investigate the solubility of the secondary metabolites present into leaves and flowers of *M. sylvestris*. The total amount of phenolic compounds and flavonoids was measured spectrophotometrically. In leaves, the level of both these classes of compounds was similar in W, 50%WE, and 70%WE samples, while it decreased significantly in the E preparation (Fig. 1A, B). A similar trend was observed for flowers (Fig. 1C, D), although flavonoids reached the maximum yield in 50WEF. These results can depend on two factors: the polarity of the solvents and the solubility rate of the plant molecules according to their chemical nature. Certainly, our data indicate that a great fraction of the phytocomplex becomes not soluble in presence of elevated concentrations of ethanol. Although this tendency could be expected for phenolics, the major hydrophobicity of flavonoids would suggest a better resuspension of them in 70%WE and/or E, compared to W. However, it is possible that common mallow flavonoids exist essentially as glycosylated forms, explaining the previous evidence. Moreover, the presence of water during maceration would support the extraction of polyphenols with high molecular weight [44, 45]. Worthy of note is the fact that the levels of phenols and flavonoids appeared to be more than ten-fold higher in the extracts isolated from leaves than flowers; a phenomenon already documented in literature [46, 47] and likely due to a typical accumulation of non-vexillary metabolites in the common mallow leaves.

Considering that *M. sylvestris* is usually consumed as an aqueous decoction or as fresh material and that ethanol determines toxicity in the human body, we decided to dry out totally our hydroalcoholic plant extracts, to resuspend them in pure ddH_2_O (aq extracts), and finally to perform spectrophotometric analyses for evaluating the residual compounds preserved in these more biocompatible preparations. Total phenols and flavonoids for all aq extracts followed the same trend shown by the pure extracts (Fig. 1E-H). In detail, the concentration of flavonoids in leaves (L aq; Fig. 1F) and phenols in flowers (F aq; Fig. 1G) were perfectly in line with those detected in the respective pure samples (Fig. 1B, C), while the levels of phenolics in the leaf aq extracts (Fig. 1E) appeared lower than its pure counterpart (Fig. 1A). This effect might be linked to the fact that a significant portion of L phenols was soluble only in the organic fraction of the solvent. By contrast, the accumulation of polyphenols in aq F samples compared to their original ones (Fig. 1H vs D) was probably due to the increased solubility of these metabolites (which probably remained aggregated in presence of ethanol), confirming our previous glycosylation theory. In addition, this datum could be justified by two other elements: the presence of anthocyanins or other water-soluble pigments in the petals of the common mallow and the capacity of polyphenols to bind proteins and generate insoluble complexes [48, 49].

An HPLC–DAD analysis was carried out to further characterize *M. sylvestris* pure and aqueous extracts, detecting and quantifying 20 plant metabolites (Fig. 1I, J; Tables 1 and 2). All the selected molecules were found at least in one sample of leaves and flowers, confirming their ubiquitous nature. Quercetin-3-glucoside was the most abundant compound identified in L pure hydroalcoholic extracts (i.e., 50WEL and 70WEL), reaching the levels of 146.9 and 159.08 ng per mg FMW respectively, while F pure hydroalcoholic extracts (i.e., 50WE, 70WE, and E) were particularly typified by Rosmarinic acid (33.09, 49.15, and 30.42 ng per mg FMW, respectively).

**Table 1:** HPLC–DAD analysis. Results of *M. sylvestris* leaf extracts expressed as ng per mg of FMW

Chemical compound	WL^*^	50WEL	50WEL aq	70WEL	70WEL aq	EL	EL aq
Gallic acid	0.36 ± 0.02	0.04 ± 0.002	10.03 ± 0.05	0.3 ± 0.001	2.51 ± 0.012	n.d.	1.39 ± 0.07
3-Hydroxytyrosol	0.042 ± 0.02	n.d.	5.5 ± 0.03	n.d.	1.84 ± 0.01	n.d.	n.d.
4-Hydroxybenzoic acid	1.27 ± 0.06	5.36 ± 0.03	6.71 ± 0.03	1.25 ± 0.006	0.53 ± 0.003	n.d.	0.52 ± 0.003
Chlorogenic acid	4.2 ± 0.0.21	0.05 ± 0.0002	6.57 ± 0.03	n.d.	0.16 ± 0.006	2.18 ± 0.01	n.d.
Caffeic Acid	0.1 ± 0.0001	n.d.	0.12 ± 0.001	11.3 ± 0.06	0.91 ± 0.004	2.52 ± 0.01	0.75 ± 0.004
Vanillic acid	0.2 ± 0.001	n.d.	0.27 ± 0.001	n.d.	2.02 ± 0.01	n.d.	1.66 ± 0.01
Epicatechin	39.3 ± 0.19	n.d.	4.98 ± 0.02	59.2 ± 0.3	2.42 ± 0.01	13.18 ± 0.06	n.d.
Syringic acid	0.01 ± 0.00005	29.14 ± 0.14	0.3 ± 0.001	56.37 ± 0.3	1.9 ± 0.001	0.19 ± 0.01	0.15 ± 0.001
*p*-Coumaric acid	0.27 ± 0.014	3.82 ± 0.2	0.28 ± 0.001	2.43 ± 0.01	0.35 ± 0.002	0.24 ± 0.01	0.26 ± 0.001
Salicylic acid	2.96 ± 0.01	33.79 ± 0.12	2.82 ± 0.01	32.8 ± 0.1	n.d.	3.5 ± 0.02	n.d.
Quercetin-3-glucoside	3.91 ± 0.02	146.9 ± 0.73	2.81 ± 0.01	159.8 ± 0.8	0.67 ± 0.03	27.03 ± 0.13	0.83 ± 0.004
Myricetin	0.53 ± 0.003	0.29 ± 0.0014	1.41 ± 0.01	6.5 ± 0.03	14.5 ± 0.07	0.62 ± 0.003	0.87 ± 0.004
Rosmarinic acid	0.21 ± 0.001	0.11 ± 0.0005	0.56 ± 0.003	6.6 ± 0.03	5.76 ± 0.03	0.25 ± 0.001	0.34 ± 0.002
Quercetin	1.9 ± 0.01	6.18 ± 0.03	4.62 ± 0.02	5.1 ± 0.03	3.39 ± 0.02	0.78 ± 0.004	1.68 ± 0.01
5,7-Dimethoxycoumarin	5.51 ± 0.03	15.32 ± 0.07	0.59 ± 0.003	16.5 ± 0.08	1.14 ± 0.06	9.54 ± 0.05	n.d.
Genistein	n.d.	n.d.	2.59 ± 0.01	1.2 ± 0.006	2.01 ± 0.01	n.d.	0.23 ± 0.001
Kaempferol	n.d.	0.78 ± 0.004	1.25 ± 0.01	1.9 ± 0.01	2.5 ± 0.01	2.15 ± 0.01	1.23 ± 0.006
1,1-Dimethylallyl caffeate	0.01 ± 0.005	0.54 ± 0.0003	0.035 ± 0.002	0.15 ± 0.001	0.28 ± 0.001	1.63 ± 0.01	0.28 ± 0.001
Chrysin	n.d.	0.21 ± 0.001	0.1 ± 0.0005	0.16 ± 0.001	0.01 ± 0.005	0.22 ± 0.001	0.1 ± 0.0005
Caffeic acid phenethyl ester	n.d.	0.35 ± 0.002	0.55 ± 0.003	0.29 ± 0.001	0.04 ± 0.002	3.0 ± 0.01	0.37 ± 0.002

^*^Legend—*W* 100% ddH_2_O, *50WE* 50% ethanol, *70WE* 70% ethanol, *E* 95% ethanol, *L* leaf, *aq* aqueous extract, *n.d.* not detected

**Table 2:** HPLC–DAD analysis. Results of *M. sylvestris* flower extracts expressed as ng per mg of FMW

Chemical compound	WF^*^	50WEF	50WEF aq	70WEF	70WEF aq	EF	EF aq
Gallic acid	0.08 ± 0.0004	n.d.	4.45 ± 0.02	n.d.	9.55 ± 0.05	1.29 ± 0.006	9.93 ± 0.05
3-Hydroxytyrosol	0.24 ± 0.001	4.32 ± 0.02	22.88 ± 0.1	3.28 ± 0.02	8.39 ± 0.04	n.d.	7.37 ± 0.36
4-Hydroxybenzoic acid	0.32 ± 0.001	n.d.	4.62 ± 0.02	n.d.	1.74 ± 0.08	n.d.	0.7 ± 0.004
Chlorogenic acid	n.d.	n.d.	5.87 ± 0.03	0.49 ± 0.002	2.69 ± 0.01	n.d.	1.88 ± 0.01
Caffeic Acid	4.42 ± 0.02	4.32 ± 0.02	0.64 ± 0.003	3.5 ± 0.02	0.48 ± 0.002	n.d.	0.67 ± 0.003
Vanillic acid	9.82 ± 0.05	6.89 ± 0.03	14.2 ± 0.007	7.77 ± 0.04	1.07 ± 0.005	n.d.	1.49 ± 0.007
Epicatechin	n.d.	n.d.	14.8 ± 0.007	n.d.	42.88 ± 0.2	n.d.	0.63 ± 0.003
Syringic acid	0.1 ± 0.0005	0.82 ± 0.004	10.94 ± 0.05	2.59 ± 0.012	3.42 ± 0.01	1.94 ± 0.0004	0.28 ± 0.001
*p*-Coumaric acid	0.5 ± 0.002	n.d.	1.49 ± 0.007	0.47 ± 0.002	3.26 ± 0.02	0.08 ± 0.0004	0.61 ± 0.003
Salicylic acid	n.d.	n.d.	1.81 ± 0.01	n.d.	5.15 ± 0.02	0.66 ± 0.003	1.48 ± 0.007
Quercetin-3-glucoside	n.d.	n.d.	2.42 ± 0.01	0.36 ± 0.002	n.d.	n.d.	2.15 ± 0.012
Myricetin	n.d.	14.49 ± 0.07	0.6 ± 0.003	12.16 ± 0.06	0.26 ± 0.001	10.25 ± 0.05	15.82 ± 0.08
Rosmarinic acid	n.d.	33.09 ± 0.2	0.24 ± 0.001	49.15 ± 0.2	10.85 ± 0.05	30.42 ± 0.15	0.88 ± 0.004
Quercetin	n.d.	1.5 ± 0.007	2.79 ± 0.01	0.87 ± 0.004	4.1 ± 0.02	1.49 ± 0.007	2.1 ± 0.01
5,7-Dimethoxycoumarin	0.01 ± 0.00005	1.75 ± 0.009	3.39 ± 0.02	1.9 ± 0.01	n.d.	n.d.	2.88 ± 0.014
Genistein	0.37 ± 0.001	0.56 ± 0.003	4.99 ± 0.02	1.47 ± 0.007	n.d.	0.33 ± 0.002	4.28 ± 0.02
Kaempferol	n.d.	24.04 ± 0.1	0.56 ± 0.003	17.01 ± 0.08	7.73 ± 0.04	13.87 ± 0.07	0.41 ± 0.002
1,1-Dimethylallyl caffeate	n.d.	n.d.	1.24 ± 0.006	0.34 ± 0.002	1.53 ± 0.007	0.19 ± 0.001	0.63 ± 0.003
Chrysin	0.32 ± 0.001	0.23 ± 0.001	0.34 ± 0.002	0.23 ± 0.001	0.18 ± 0.001	0.14 ± 0.001	0.22 ± 0.0001
Caffeic acid phenethyl ester	n.d.	1.17 ± 0.006	1.41 ± 0.007	1.14 ± 0.006	n.d.	1.23 ± 0.006	0.3 ± 0.001

^*^Legend—*W* 100% ddH_2_O, *50WE* 50% ethanol, *70WE* 70% ethanol, *E* 95% ethanol, *F* flower, *aq* aqueous extract, *n.d.* not detected

Even though only one portion of the phytocomplex was defined in this chromatographic analysis, results showed that L samples were richer in phenols and flavonoids than F ones, as already evidenced through the previous spectrophotometric measurements. Remarkably, some chemical compounds appeared undetectable in the pure hydroalcoholic extracts but were present in the respective aq samples, and vice versa (although in lesser size). Obviously, this phenomenon might be linked with a different solubility of the plant metabolites between water and ethanol, also explaining the increase of total phenolic and flavonoid compounds estimated by spectrophotometric assays in the aq extracts. In general, L and F 50WE and 70WE macerations revealed the highest contents of the series for the chosen secondary metabolites, indicating that these two extraction procedures were the most profitable in terms of yield and composition. Indeed, it is widely documented that organic solvents are poor diluents for phenolics and flavonoids if not mixed with a certain percentage of water [50–52].

As *M. sylvestris* is known to contain anthocyanins [53], we decided to estimate the level of these phytochemicals in all our extracts, in order to reach a higher level of molecular characterization. To do it, we applied a spectrophotometric method exploiting the peculiar absorption range of the different anthocyanins (i.e., from 490 to 550 nm) [54]. No anthocyanin was recorded in the L samples, while their signals were easily detectable in the F extracts (Fig. 1K, L). At this point, it is necessary to underline that the levels of total flavonoids and those of the anthocyanins would not seem to be in line, being the latter higher than the first ones. However, a reasonable explanation for this evidence could be associated to the fact that anthocyanins are prone to undergo structural transformations and complexation reactions [55] and that maybe the aluminum chloride method is mainly devoted for the quantitation of simple flavonoids. Although anthocyanins are water soluble pigments, we observed that the extraction of these compounds increased when the organic solvent was added to the maceration, reaching the maximum yields with 70%WE and then decreasing with further supplementation of ethanol, as already reported by Cacace and Mazza [56]. MAL3G and PTD3G were the most abundant anthocyanins present in the petals of common mallow, followed by PLG, CYN, DEL, and PEO, in that order (Fig. 1K). It is unsurprising that PEO was detected in low concentration, considering that usually this anthocyanin is usually not typical of the common mallow [3]. In the aq extracts (Fig. 1L), a decrease of anthocyanin concentration was detected compared to the respective pure samples, although they remained stable one each other in terms of proportion. This phenomenon could be linked to degradation events occurring during the lyophilization process and/or to the reduced capacity of the new solvent (i.e., ddH_2_O) to resuspend these molecules.

All together, these metabolomic analyses allowed us to define more in detail the phytocomplex of leaves and flowers from *M. sylvestris* and to investigate the solubilization properties of plant secondary metabolites in different extraction solvents.

### MiRNome analysis

**Fig. 2:**
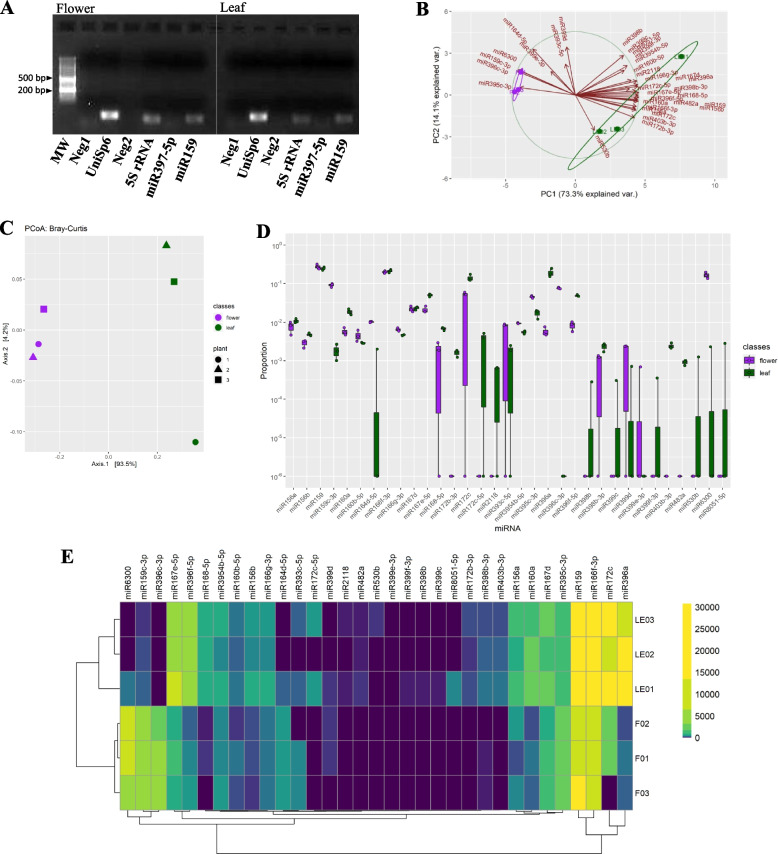
MiRNome analysis. **A** Validation of presence and extraction procedure for plant miRNAs. After miRNA isolation from *M. sylvestris* flowers (left panel) and leaves (right panel) and retrotranscription reaction, PCR amplifications were carried out and the relative products fractioned on agarose gel and visualised under UV-light. The signals for the UniSp6 (the positive control of the retrotranscription kit), the plant 5S rRNA, and one out of two selected ubiquitous plant miRNAs (i.e., miR397-5p, and miR159) are shown. In addition, the absence of amplicons in the lanes of the negative controls (Neg 1 and Neg 2; see Materials and Methods section) can be noticed. (MW, molecular weight). The full-length image of the gel is reported in Supplemental material 3. **B** PCA graph based on presence and abundances of the miRNAs detected in the common mallow leaves (L, green) and flowers (F, violet). Red arrows indicate the effect of each miRNA on the first (PC1) and second (PC2) component. **C** PCoA graph based on the frequency of miRNAs present in *M. sylvestris* leaves (green spots) and flowers (violet spots). **D** Graph of the differential abundance for the miRNAs found in leaves (green bars) and flowers (violet bars) of the common mallow. The relative abundance is plotted in log10 on the y-axis. **E** Heatmap of the abundance of the the detected miRNAs in the different flower and leaf samples, with relative hierarchical clustering

Recently, beyond secondary metabolites, plant miRNAs have attracted interest and curiosity from the scientific community, due to their CKR capacity of the human gene expression [18–20]. Considering this premise, it would be possible to suppose that the beneficial properties of *M. sylvestris* [57] are attributable also to its content in miRNAs. Thus, first, the presence of two plant miRNAs (i.e., miR397-5p, miR159), chosen for their wide distribution in various plant species and their elevated nucleotide conservation in the plant kingdom [58, 59], was investigated (Fig. 2A). Positive (UniSp6; 5S RNA) and negative (Neg1; Neg2) controls (described in Materials and Methods section) were performed to confirm accuracy and efficiency of the extraction and detection methods. As shown in Fig. 2A, only one out of two selected plant miRNAs (i.e., miR159) was confirmed in the mallow samples (data also validated then by the NGS approach).

After this preliminary check, taking into account the potential inhomogeneity in miRNA expression linked to possible physiological and metabolic variations and to provide statically significant results, 6 pools of miRNAs were isolated from leaves (i.e., LE01, LE02, LE03) and flowers (i.e., F01, F02, F03) of different *M. sylvestris* specimens and subjected to NGS analysis. Small-RNA sequencing detected 419.188 reads mapping to all samples, for a total of 33 different miRNAs, all attributable to already known families of plant miRNAs. The distribution of the reads for each miRNA among the samples is reported in Table 3, together with their nucleotide sequences. In particular, 31 miRNAs were identified in the leaves, although only 20 present in the whole LE series, while 23 were found in the flowers, with just 17 detectable in all F specimens. Two miRNAs (i.e., miR396c-3p, miR399e-3p) characterized only the flower samples, while 12 were typical of leaves (i.e., miR3600, miR159c-3p, miR172b-3p, miR172c-5p, miR2118, miR398b, miR399c, miR399f-3p, miR403b-3p, miR482a, miR530b, miR8051-5p). The most abundant miRNAs in flowers were miR159, miR166f-p, and miR6300, while in leaves were miR167e-5p, miR159, miR166f-p, miR172c, miR396a, and miR396f-5p.

**Table 3:** Identified miRNAs. List of the expressed miRNAs detected by NGS analysis in the common mallow, together with their respective nucleotide sequence. The number of raw sequencing reads counted for each miRNA in the samples of leaves (i.e., LE01, LE02, LE03) and flowers (i.e., F01, F02, F03) were indicated

miRNA	Sequence (5’-3’)	LE01	LE02	LE03	F01	F02	F03
miR156a	UGACAGAAGAGAGUGAGCAC	1204	803	1063	203	424	374
miR156b	UUGACAGAAGAUAGAGAGCAC	556	356	452	140	140	96
miR159	UUUGGAUUGAAGGGAGCUCUA	27,389	20,834	21,236	10,549	11,197	14,364
miR159c-3p	CUUGGACUGAAGGGAGCUCCC	221	207	87	4117	3397	4496
miR160a	UGCCUGGCUCCCUGAAUGCCA	2278	1706	1341	317	198	237
miR160b-5p	UGCCUGGCUCCCUGUAUGCCG	348	222	258	270	135	194
miR164d-5p	UGGAGAAGCAGGGCACGUGCA	249	n.d.^*^	n.d.	450	445	442
miR166f-3p	UCGGACCAGGCUUCAUUCCCU	23,685	17,879	18,657	8694	7830	9679
miR166g-3p	UCUCGGACCAGGCUUCAUUCC	556	368	408	314	265	266
miR167d	UGAAGCUGCCAGCAUGAUCUUA	3031	1613	2078	1132	796	944
miR167e-5p	UGAAGCUGCCAGCAUGAUCUA	6638	3461	4380	1133	797	837
miR168-5p	UCGCUUGGUGCAGGUCGGGAA	896	547	524	129	80	n.d.
miR172b-3p	AGAAUCUUGAUGAUGCUGCAU	153	133	160	n.d.	n.d.	n.d.
miR172c	UGAAUCUUGAUGAUGCUACGC	15,076	9785	14,957	2587	2172	n.d
miR172c-5p	AGCAUCUUCAAGAUUCACA	484	n.d.	442	n.d.	n.d.	n.d.
miR393c-5p	UCCAAAGGGAUCGCAUUGAUC	231	n.d.	215	394	n.d.	373
miR395c-3p	CUGAAGUGUUUGGGGGAACUC	1480	1504	1695	1816	2047	2050
miR396a	UUCCACAGCUUUCUUGAACUG	30,763	13,649	13,059	335	198	226
miR396c-3p	GUUCAAUAAAGCUGUGGGAAG	n.d.	n.d.	n.d.	3201	3183	3607
miR396f-5p	UUCCACAGCUUUCUUGAACUU	6359	3658	4000	385	241	463
miR398b	UGUGUUCUCAGGUCACCCCU	35	n.d.	n.d.	n.d.	n.d.	n.d.
miR398b-3p	UGUGUUCUCAGGUCGCCCCUG	316	211	146	59	n.d	55
miR399c	CGCCAAAGGAGAGUUGCCCUG	38	n.d.	n.d.	n.d.	n.d.	n.d.
miR399d	UGCCAAAGGAGAGUUGCCCUG	88	n.d.	n.d.	106	100	n.d.
miR399e-3p	UGCCAAAGGAGAUUUGCCCGG	n.d.	n.d.	n.d.	30	n.d.	n.d.
miR399f-3p	CGCCAAAGGAGAAUUGCCCUG	44	n.d.	n.d.	n.d.	n.d.	n.d.
miR403b-3p	UUAGAUUCACGCACAAACUCG	264	226	190	n.d.	n.d.	n.d.
miR482a	UCUUUCCUACUCCUCCCAUUCC	118	79	64	n.d.	n.d.	n.d.
miR530b	UGCAUUUGCACCUGCACCUUA	n.d.	n.d.	108	n.d.	n.d.	n.d.
miR2118	UUGCCGAUUCCACCCAUUCCUA	82	n.d.	55	n.d.	n.d.	n.d.
miR3954b-5p	UUAGAUUCACGCACAAACUCG	690	384	512	401	395	444
miR6300	GUCGUUGUAGUAUAGUGG	287	n.d.	n.d.	7083	8385	6150
miR8051-5p	UAGUAUGGUAGAAAGAUUCA	350	n.d.	n.d.	n.d.	n.d.	n.d.

^*^n.d. not detected

To explore the diversity of the miRNA presence and frequency between leaves and flowers, we performed a PCA (Fig. 2B). The two principal components (PC1 and PC2) together explained 87.4% of the whole dataset variance, that is they were able to separate clearly and group the detected miRNAs in two main clusters, according to their flower or leaf origin. We confirmed this evidence also by carrying out a PCoA (Fig. 2C), a method which considers the dissimilarities among data and not their similarities like in the case of PCA. Furthermore, the distance matrix used as input for the PCoA was employed to assess the statistical significance of the two clusters. In detail, the matrix was evaluated by using the PERMANOVA test. This analysis allowed to verify the predictive effect of the presence for each miRNA on the basis of the plant tissue (i.e., flower vs. leaf) and/or the investigated specimen (i.e., 01 vs. 02 vs. 03), through the calculation of the relative pseudoF, R^2^, and *p*-values. Our results evidenced that the localization in plant was the only parameter able to significantly predict for miRNAs’ presence, explained the 91% of the variance.

Small RNA-seq outcomes were normalized and analyzed using the DESeq2 R package, to investigate more in detail the validity of our data. The abundance of all miRNAs in flowers and leaves was reported, although only 16 of them significantly differed between the two plant organs. In detail, 8 miRNAs typified the leaves (i.e., miR396a, miR403b-3p, miR396f-5p, miR172b-3p, miR482a, miR160a, miR167e-5p, miR172c-5p), while other 8 the flowers (i.e., miR396c-3p, miR159c-3p, miR6300, miR3954b-5p, miR395c-3p, miR160b-5p, miR166g-3p, miR164d-5p). The visualization of the expression levels of all miRNAs was reported in Fig. 2D. The results relative to miRNAs’ frequency were also shown as a heatmap (Fig. 2E), where the colors provide a rapid information about the differences found in their abundance in the whole studied series. The hierarchical clustering deriving from the heatmap corroborated our previous suppositions.

It is important to remember that miRNAs play crucial roles in plants, modulating their propagation, reproduction, development, growth, homeostasis, signaling, response to biotic and abiotic stressors, etc. [60]. Moreover, it has been documented that the transcriptional patterns of these small nucleic acids are finely regulated and dependent on time (i.e., life phase), space (i.e., tissue), and exogenous stimuli (e.g., temperature variation) [61–63]. Therefore, a bioinformatics analysis was carried out to identify the putative targets of the miRNAs detected in common mallow, to understand their potential functions in the plant and clarify if their expression profiles could vary according to the tissue (i.e., leaf, flower) in which they were synthesized.

The list of the plant mRNA targets predicted for each *M. sylvestris* miRNA was reported in detail in Supplemental material 1. However, to make all this information more usable to the reader, the most significant results were summarized in Table 4. Overall, 19 miRNAs out of 33 presented as targets at least one transcript encoding for chromosome scaffold proteins (CSSs), which are responsible for holding the chromatin in compact form [64]. Although the condensed structure of heterochromatin mainly depends on histone-related proteins [65], it is possible to hypothesize that also CSSs may be involved in the regulation of DNA close conformation [66]. Therefore, the miRNAs potentially able to modulate CSSs’ mRNAs could exert a key role in the gene expression of *M. sylvestris*, acting as epigenetic mediators. Examples might be miR398b, miR399c, miR403b-3p, and miR530b.

**Table 4:** Summary of the bioinformatics prediction against plant transcriptome. For each investigated miRNA (Query), the putative plant target mRNAs showing an expectation (Exp) ≤ 1 (where possible, otherwise the lowest registered values) were listed and described as Unigene-DFCI Gene Index-GORAGI IDs (Accession) and in full (Description). The complete output of this bioinformatics analysis was reported in Supplemental material 1

Query	Exp	Target	
		**Accession**	**Description**
**miR156a**	1.0	CO092899	Chromosome chr1 scaffold_136
	1.0	TC6174	Chromosome chr8 scaffold_34
	1.0	TC6994	Chromosome chr1 scaffold_136
**miR156b**	0.0	TC5782	Unknown protein
	0.0	TC7659	Unknown protein
**miR159**	1.0	TC9277	ATP synthase F0 subunit 6
**miR159-3p**	2.0	CO076957	Amphiregulin long form
	2.0	TC9277	ATP synthase F0 subunit 6
**miR160a**	1.0	TC8008	Chromosome chr6 scaffold_3
	1.0	TC4570	Auxin response factor 3
**miR160b-5p**	0.0	TC8008	Chromosome chr6 scaffold_3
	0.0	TC4570	Auxin response factor 3
**miR164d-5p**	1.5	CO109521	NAC domain protein NAC1
**miR166f-3p**	1.0	TC6854	Class III HD-Zip protein 4
**miR166g-3p**	2.5	CO079323	Chromosome chr14 scaffold_27
**miR167d**	3.0	TC1436	Chromosome chr5 scaffold_2
	3.0	TC3171	Zinc finger, C3HC4 type family protein
	3.0	TC484	Cytochrome P450
**miR167e-5p**	3.0	TC1436	Chromosome chr5 scaffold_2
	3.0	TC3171	Zinc finger, C3HC4 type family protein
	3.0	TC484	Cytochrome P450
**miR168-5p**	2.5	CO084924	AGO1-1
**miR172b-3p**	0.5	TC7001	Chromosome chr6 scaffold_3
	0.5	TC4318	Ethylene-responsive transcription factor related to APETALA2
	0.5	TC8328	Chromosome chr6 scaffold_3
	0.5	TC8568	Chromosome chr13 scaffold_17
**miR172c**	2.5	TC7001	Chromosome chr6 scaffold_3
	2.5	TC7575	Chromosome chr7 scaffold_20
	2.5	TC4318	Ethylene-responsive transcription factor related to APETALA2
	2.5	TC8568	Chromosome chr13 scaffold_17
**miR172c-5p**	3.0	TC2843	Plastid division protein
	3.0	CO097477	NADPH-cytochrome P450 oxydoreductase isoform 3
	3.0	TC9053	Chromosome chr7 scaffold_192
	3.0	TC4030	Chromosome chr12 scaffold_47
	3.0	TC8617	Glycosyl hydrolase
	3.0	TC7382	Chromosome chr18 scaffold_1
	3.0	TC3352	Chromosome undetermined scaffold_670
	3.0	TC8563	Chromosome chr4 scaffold_83
**miR393c-5p**	1.0	TC7732	Transport inhibitor response 1
**miR395c-3p**	2.5	TC7582	Pyruvate decarboxylase
**miR396a**	2.5	TC9189	Chromosome chr2 scaffold_132
**miR396c-3p**	3.5	TC4016	Chromosome chr5 scaffold_64
	3.5	TC5991	Calcium binding protein
	3.5	TC9308	Chromosome chr19 scaffold_4
	3.5	TC21	Unknown protein
	3.5	CO099385	Chromosome chr3 scaffold_8
	3.5	CO128430	Chromosome chr17 scaffold_16
	3.5	CO129875	Chromosome chr17 scaffold_12
	3.5	TC1113	Chromosome undetermined scaffold_254
**miR396f-5p**	2.5	TC9189	Chromosome chr2 scaffold_132
**miR398b**	3.0	CO114193	Chromosome chr1 scaffold_5
**miR398b-3p**	2.5	CO105378	RING-H2 finger protein ATL3F
**miR399c**	2.5	CO072266	Unknown protein
	2.5	CO092308	Chromosome chr17 scaffold_12
	2.5	CO120548	Chromosome chr4 scaffold_443
**miR399d**	1.5	CO072266	Unknown protein
**miR399e-3p**	2.0	TC7413	Chromosome chr11 scaffold_13
	2.0	CO124179	MYB transcription factor
**miR399f-3p**	3.0	CO095104	ATP-dependent Clp protease proteolytic subunit
	3.0	CO095019	Os11g0104900 protein
**miR403b-3p**	3.0	TC2550	Chromosome chr5 scaffold_124
**miR482a**	2.5	CO081126	ATPase
**miR530b**	2.0	TC2909	Chromosome chr6 scaffold_3
**miR2118**	1.0	TC5359	Cytochrome b
**miR3954b-5p**	3.0	TC2550	Chromosome chr5 scaffold_124
**miR6300**	2.0	TC6704	Unknown protein
**miR8051-5p**	2.5	CO082018	Chromosome chr4 scaffold_6
	2.5	CO127801	Isoform 2 of Q8LPQ8

Regarding the miRNAs found only in the leaves, particular targets were predicted for miR172b-3p, miR172c-5p, miR482a, and miR2118. In detail, the first one would seem to bind and modulate the Ethylene-responsive transcription factors related to APETALA2. As these proteins are involved in several processes (e.g., primary and secondary metabolism, growth programs, response to environmental stimuli) and can act both as activators and repressors of the transcription [67], it is complicated to define the specific role of miR172b-3p in our case. By contrast, the synthesis of miR172c-5p, miR482a, and miR2118 in LE samples suggested a fine regulation of the mitochondrial and plastid functions, since their putative targets were linked to plastid division, cytochrome activity, ATP degradation, and hydrolysis of complex sugars [68, 69].

The two miRNAs typifying the flowers of common mallow, that is miR396c-3p and miR399e-3p, were found to potentially interact with Calcium binding protein and MYB transcription factor, in that order, excluding Chromosome scaffold proteins (already discussed above). Literature reports that calcium and MYB transcription factor are related to flower development and color [70–72]; therefore, our results would suggest that these factors were probably no more necessary in the analysed flowers (so, to be down-regulated), as already completely mature.

In general, at this stage, we can only suppose the plant functions in which the detected miRNAs are involved because further molecular investigations specifically focused on each one of them are necessary to clearly understand their role. However, the demonstration of their existence in the common mallow might open new perspectives in the study of the gene regulation for this plant species.

Despite some scholars believe plant miRNAs cannot be absorbed by diet and ascribe the evidence of their presence in human samples to artifacts or contamination [73–75], a great part of the scientific community has proved that food and medicinal plant-derived miRNAs can be acquired and transported in the bloodstream, released in specific human tissues, and even capable to perform CKRs of the hosts’ gene expression [18–21]. This testimony suggests that plant miRNAs may play a key role in modulating human health in physiological and pathological states. In this regard, in COVID time, ginger and grapefruit miRNAs have been even proposed as possible antiviral agents, potentially targeting SARS-CoV-2 genes [76].

**Fig. 3:**
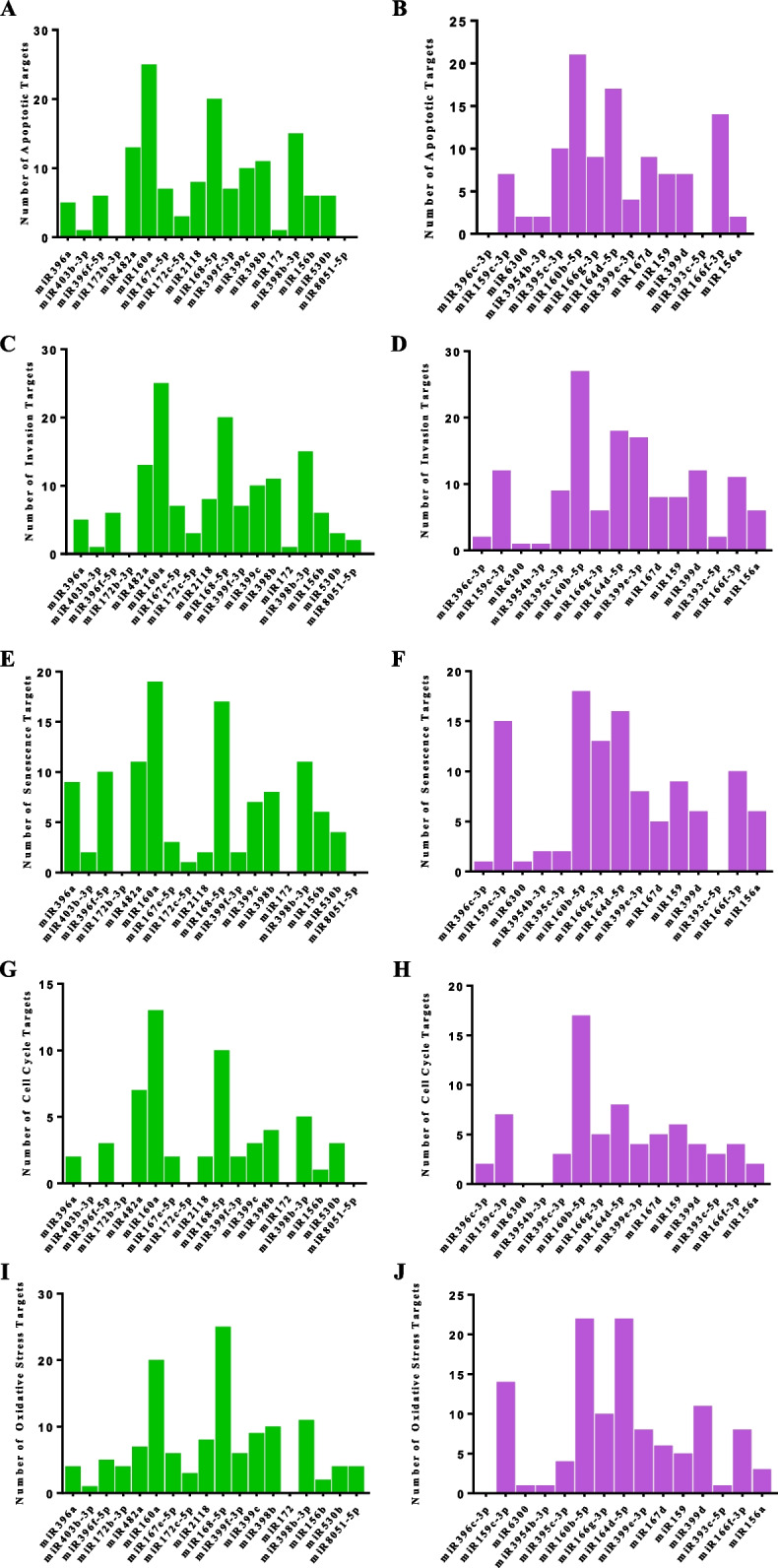
Human target prediction. Number of the total putative human mRNA targets predicted for each miRNA present in the common mallow leaf (green graphs) and flower (violet graphs), grouped for cell biological processes (**A** and **B**: Apoptosis; **C** and **D**: Invasion; **E** and **F**: Senescence; **G** and **H**: Cell Cycle; **I** and **J**: Oxidative stress). Data elaborated from the information reported in Supplemental material 2

Considering the previous premise, the last step of our work consisted in the bioinformatics prediction of the putative human targets, involved in 5 fundamental mammalian cellular processes (i.e., apoptosis, senescence, cell-cycle, oxidative stress, and invasiveness), for the 33 miRNAs sequenced from common mallow. The miRNA target prediction tool used in this work [39, 40] identified, in the human transcriptome, a total of 383 mRNAs that theoretically could be bound and regulated by *M. sylvestris* miRNAs (Supplemental material 2). Only two miRNAs (i.e., miR530b and miR8051-5p) did not show any human target at high prediction efficiency. To resume the huge amount of data obtained by this analysis, the three most significant human targets for each miRNA were reported in Table 5. In addition, the whole set of predictions were schematized in the graphs shown in Fig. 3, where the number of putative human transcripts was indicated for the most significantly expressed miRNAs in leaves and flowers. Based on this bioinformatics clustering, miR160a, miR168-5p, and miR398b-3p isolated from leaves and miR160b-5p and miR164d-5p from flowers presented the highest number of targets for the analysed pathways. Interestingly, miR172b-3p from leaves showed only targets linked to the oxidative stress, while miR396c-3p and miR6300 from flowers revealed the lowest number of total targets, exactly 5 per each.

**Table 5:** Summary of the bioinformatics prediction against human transcriptome. For each investigated miRNA (Query), the most significant putative human target mRNAs, showing at least a percentage of maximum likelihood > 0.86, were listed and described as NCBI acronyms (Code) and in full (Description). The complete output of this bioinformatics analysis was reported in Supplemental material 2

Query	Target	
	**Code**	**Description**
**miR156a**	DCC	Netrin receptor DCC
	IGFBP3	Insulin-like growth factor-binding protein 3
	PRNP	Major prion protein
**miR156b**	IGFBP3	Insulin-like growth factor-binding protein 3
	HGF	Hepatocyte growth factor receptor
	OXR1	Oxidation resistance protein 1
**miR159**	CTSK	Cathepsin K
	BCL2L1	Bcl-2-like protein 1
	CDKN1B	Cyclin-dependent kinase inhibitor 1B
**miR159c-3p**	TRAF2	TNF receptor-associated factor 2
	CDKN2D	Cyclin-dependent kinase 4 inhibitor D
	MTA1	Metastasis-associated protein 1
**miR160a**	AKT1	RAC-alpha serine/threonine-protein kinase
	CDKN2A	Cyclin-dependent kinase inhibitor 2A
	E2F4	Transcription factor E2F4
**miR160b-5p**	BOK	Bcl-2-related ovarian killer protein
	CDK2	Cyclin-dependent kinase 2
	MPV17	Protein Mpv17
**miR164d-5p**	STAT5A	Signal transducer and activator of transcription 5A
	CEACAM1	Carcinoembryonic antigen-related cell adhesion molecule 1
	EPX	Eosinophil peroxidase
**miR166f-3p**	IRF3	Interferon regulatory factor 3
	AVEN	Cell death regulator Aven
	MMP14	Matrix metalloproteinase-14
**miR166g-3p**	HDAC6	Histone deacetylase 6
	GPX3	Glutathione peroxidase 3
	CDK2	Cyclin-dependent kinase 2
**miR167d**	NFKB1	Nuclear factor NF-kappa-B p105 subunit
	KISS	Metastasis-suppressor KiSS-1
	SRXN1	Sulfiredoxin-1
**miR167e-5p**	TNFSF11	Tumor necrosis factor receptor superfamily member
	GSK3B	Glycogen synthase kinase-3 beta
	MGAT5	Alpha-1,6-mannosylglycoprotein 6-beta-N-acetylglucosaminyltransferase
**miR168-5p**	TRAF2	TNF receptor-associated factor 2
	KISS1R	KiSS-1 receptor
	APOE	Apolipoprotein E
**miR172b-3p**	FASLG	FASLG receptor
	REL	Proto-oncogene c-Rel
	GSR	Glutathione reductase
**miR172c**	FASLG	FASLG receptor
	CTBP1	C-terminal-binding protein 1
**miR172c-5p**	TRAF3	NF receptor-associated factor 3
	IGF1	Insulin-like growth factor I
	PXDN	Peroxidasin homolog
**miR393c-5p**	MMP9	Matrix metalloproteinase-9
	GSK3B	Glycogen synthase kinase-3 beta
	EPX	Eosinophil peroxidase
**miR395c-3p**	RET	Retinol-binding protein
	TNFRSF1A	Tumor necrosis factor receptor type 1-associated DEATH domain protein
	MCAM	Cell surface glycoprotein MUC18
**miR396a**	CTNNA1	Catenin alpha-1
	TNFRSF1B	TNF receptor-associated factor 2
	NFKB1	Nuclear factor NF-kappa-B p105 subunit
**miR396c-3p**	RBL1	Retinoblastoma-like protein 1
	HDAC2	Histone deacetylase 2
	HDAC1	Histone deacetylase 2
**miR396f-5p**	DFFA	NA fragmentation factor subunit alpha
	ANGPTL7	Angiopoietin-related protein 7
	CCNB1	G2/mitotic-specific cyclin-B1
**miR398b**	SOCS3	Suppressor of cytokine signaling 3
	ERB2	Estrogen receptor b2
	CDK4	Cyclin-dependent kinase 4
**miR398b-3p**	TP53I3	TP53I3 protein
	PRKCD	Protein kinase C delta type
	ERB2	Estrogen receptor b2
**miR399c**	MMP9	Matrix metalloproteinase-9
	FASLG	FASLG receptor
	GSR	Glutathione reductase
**miR399d**	LAMB1	Laminin subunit beta-1
	BOK	Bcl-2-related ovarian killer protein
	GSR	Glutathione reductase
**miR399e-3p**	TIMP1	Metalloproteinase inhibitor 1
	CCS	Copper chaperone for superoxide dismutase
	IRF3	Interferon regulatory factor 3
**miR399f-3p**	MMP9	Matrix metalloproteinase-9
	CASP8	Caspase-8
	LPO	Lactoperoxidase
**miR403b-3p**	IGF1R	Insulin-like growth factor 1 receptor
	CASP8	Caspase-8
	STAT5A	Signal transducer and activator of transcription 5A
**miR482a**	NOS2	Nitric oxide synthase, inducible
	CDKN1A	Cyclin-dependent kinase inhibitor 1
	SERPINE1	Plasminogen activator inhibitor 1 RNA-binding protein
**miR2118**	CCS	Copper chaperone for superoxide dismutase
	RELA	Bifunctional (p)ppGpp synthase/hydrolase RelA
	CXCR4	C-X-C chemokine receptor type 4
**miR3954b-5p**	STAT5A	Signal transducer and activator of transcription 5A
	IGF1R	Insulin-like growth factor 1 receptor
	CASP8	Caspase-8
**miR6300**	DUOX1	Dual oxidase 1
	TPBG	Trophoblast glycoprotein
	IFNG	Interferon gamma

**Fig. 4:**
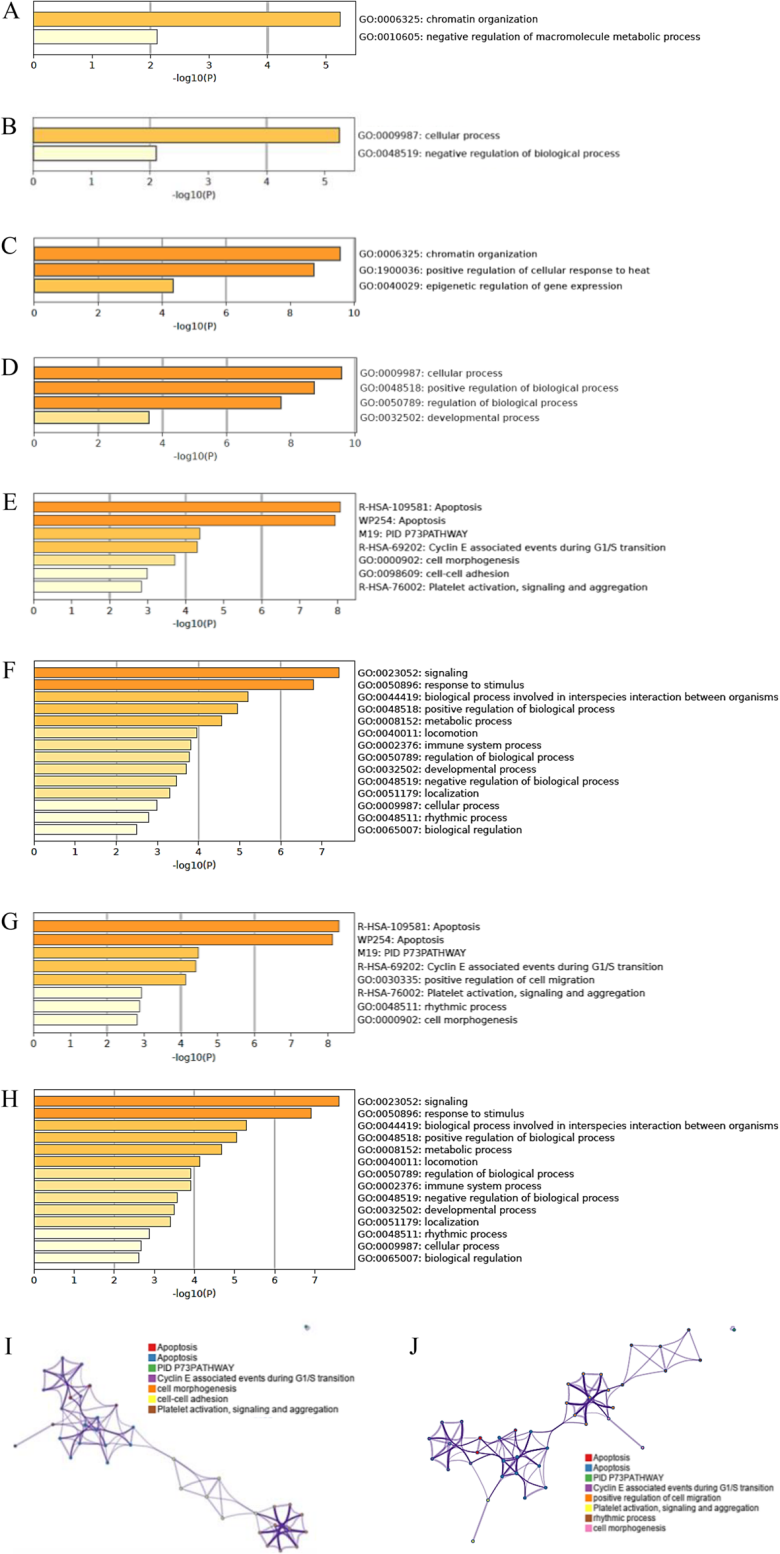
Gene Ontology (GO) enrichment analyses. **A** Bar graph of plant GO enriched terms across input gene lists relative to leaf miRNAs’ putative plant targets. **B** Top-level plant GO biological processes relative to GO enriched terms reported in panel (**A**). **C** Bar graph of plant GO enriched terms across input gene lists relative to flower miRNAs’ putative plant targets. **D** Top-level plant GO biological processes relative to GO enriched terms reported in panel (**C**). **E** Bar graph of human GO enriched terms across input gene lists relative to leaf miRNAs’ putative human targets. **F** Top-level human GO biological processes relative to GO enriched terms reported in panel (**E**). **G)** Bar graph of human GO enriched terms across input gene lists relative to flower miRNAs’ putative human targets. **H** Top-level human GO biological processes relative to GO enriched terms reported in panel (**G**). **I** Network of GO enriched terms relative to the analysis reported in panel (**E**) and (**F**). **J)** Network of GO enriched terms relative to the analysis reported in panel (**G**) and (**H**). In the bar graphs the colours indicate different *p*-values. In the networks, cluster IDs are coloured in different ways, nodes that share the same cluster ID are typically close, and the colour intensity of the conjunctions indicate different *p*-value (i.e., terms containing more genes tend to have a major significance)

In order to identify the main biological processes potentially linked to the whole set of putative targets for *M. sylvestris* miRNAs, GO enrichment analyses were carried out (Fig. 4). The targets predicted for leaf miRNAs appeared in their entirety able to act on chromatin organization and to negatively influence metabolic and cellular processes in plants (Fig. 4A, B). In parallel, always in plants, the profile of the transcripts modulated by the flower miRNAs converged on regulative mechanisms for chromatin arrangement, epigenetics, development, and response to heat (Fig. 4C, D). These results were quite expected because, as already previously stated, miRNAs would seem to modulate plant gene expression also influencing the heterochromatin/euchromatin ratio. Interestingly, the biological function associated to the temperature variation (i.e., GO:1,900,036) could suggest that flowers were living a change of the environmental condition of their habitat at the sampling time. On the other hand, assuming the existence of the CKR phenomenon, the human GOs detected by the enrichment analysis using the leaf miRNAs’ targets as input data were listed in Fig. 4E, F, and I. In this case, apoptosis, platelet activation, and cell cycle, morphogenesis, and adhesion were the main processes predicted to be potentially controlled. A very similar prediction was also obtained considering the targets of the miRNA profile from flowers (Fig. 4G, H, J), although two new functions were detected (i.e., cell migration and rhythmic process). This last evidence would indicate that miRNAs might represent a significant bioactive component of *M. sylvestris* derivatives (e.g., decoctions, fresh and dry leaves) on human health, clarifying the medicinal role that this species has played in folk phytotherapy since time immemorial.

## Conclusions

In this paper, the metabolomic profile of the phytocomplex purified from leaves and flowers of *M. sylvestris* by different extraction solvents was characterized. Here, beyond the measurement of the total content for phenols and flavonoids, we specifically detected and quantified 26 different compounds in the common mallow extracts by HPLC–DAD and spectrophotometric analyses, highlighting that their concentration in the several samples varied according to chemical nature and type of solvent. Moreover, the capacity of the secondary metabolites isolated from this plant species to remain stable after drying out, for eliminating any trace of organic solvent (due to its intrinsic toxicity), and resuspension in pure water was evaluated. We found that *M. sylvestris* phytochemicals remained as unaltered after the application of this protocol, suggesting for them a possible use in biological/medical studies, including in vitro and in vivo experiments on mammalian systems, and a potential application as dietary supplements (e.g., antioxidants) and/or biologically active substances (e.g., antineoplastic, immunomodulant). In the second part of the research, the miRNome expressed in *M. sylvestris* leaves and flowers was analysed by NGS. For the first time in the literature, 33 miRNAs were linked to this plant species, although 10 of them were typical of leaf tissues and 2 codified only in flowers. Bioinformatics predictions were carried out to identify the putative targets for the detected miRNAs in both plant and human transcriptomes. The main results suggested a potential role for these small nucleic acids in chromatin remodelling of *M. sylvestris* cells. In addition, the possibility for them to work as cross-kingdom regulation agents in the human body was suggested, since they presented a total of 383 putative mRNA targets involved in 5 fundamental mammalian cellular processes (i.e., apoptosis, senescence, cell-cycle, oxidative stress, and invasiveness). Thus, beyond secondary metabolites, *M. sylvestris* miRNAs ingested through food or medicinal preparations could carry out specific effects on the human health, explaining the beneficial action of this herb as handled down by the folk medicine. The possibility to develop a phytotherapeutic approach based on miRNAs isolated from medicinal plants, such as the common mallow, might represent a further step for next generation gene therapies. Indeed, to date, pharmaceutical companies have focused their attention on plant secondary metabolites, excluding the concept to investigate potential biological functions at the expense of exogenous miRNAs. The phenomenon of the CKR by small RNA molecules has been detected for the first time in plants infected by viruses; therefore, why should not it be valid for plant miRNAs on human systems, considering the strong relationship that human civilizations have developed during the evolution with the components of their respective environments?

### Supplementary Information

**Additional file 1: Supplemental Material 1.** Plant target prediction for *M. sylvestris* miRNAs. For each specific miRNA, the results obtained by the PsRNAtarget software and containing the list of putative plant mRNA targets predicted by the bioinformatics analysis was provided. In detail, for each miRNA, the following information was reported: miRNA Accession; Target Accession; Expectation; UPE; miRNA start; miRNA end; Target start; Target end; miRNA aligned fragment alignment; Target aligned fragment; Inhibition; Target; Description; Multiplicity.

**Additional file 2: Supplemental Material 2.** Human target prediction for *M. sylvestris* miRNAs. For each specific miRNA, the results obtained by the PsRNAtarget software and containing the list of putative human mRNA targets predicted by the bioinformatics analysis was provided. In detail, for each miRNA, the targets, described as NCBI acronyms, and the relative percentage of maximum likelihood were listed, according to the cellular pathway in which they are involved.

**Additional file 3: Supplemental Material 3.** Full-length version of the gel shown in Fig. 2A.

## Data Availability

The datasets generated and analysed during the current study are available in the Sequence Read Archive (https://www.ncbi.nlm.nih.gov/sra; ID: PRJNA952515; submission: SUB13030104), while the discussed results are reported in the main text or in the supplementary materials of the manuscript. The plant specimens sampled in this work are preserved in the Herbarium collection at the Botanical Gardens of Rome Tor Vergata.
