# Supplementary figures and images for: Profile and potential bioactivity of the miRNome and metabolome expressed in Malva sylvestris L. leaf and flower
Source: BMC Plant Biol. 2023 Sep 19;23:439. doi: 10.1186/s12870-023-04434-1 (PMC10507896; doi:10.1186/s12870-023-04434-1)

Supplemental Material 3. Full-length version of the gel shown in Figure 2A.

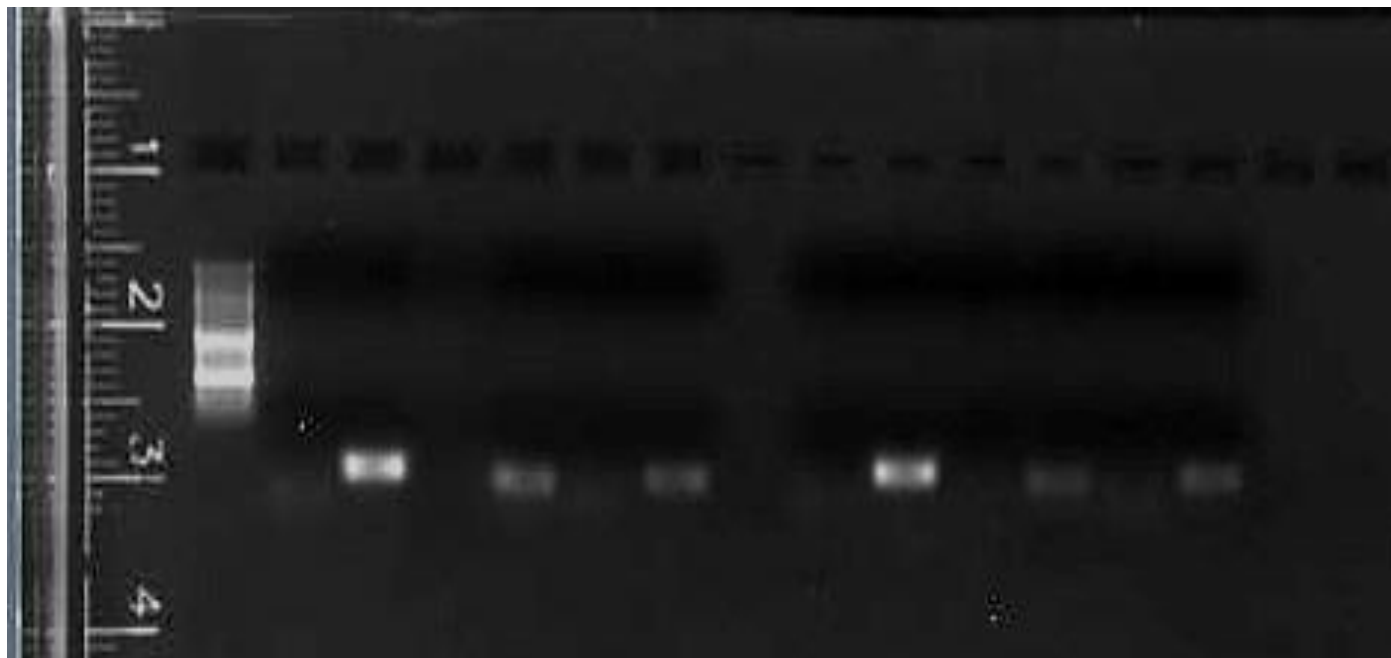

Supplement: Supplementary file 3 — Additional file 3: Supplemental Material 3. Full-length version of the gel shown in Fig. 2A. [file 12870_2023_4434_MOESM3_ESM.pdf]
